# Small non-coding RNA transcriptome of four high-altitude vertebrates and their low-altitude relatives

**DOI:** 10.1038/s41597-019-0204-5

**Published:** 2019-10-04

**Authors:** Keren Long, Siyuan Feng, Jideng Ma, Jinwei Zhang, Long Jin, Qianzi Tang, Xun Wang, Miaomiao Mai, Weihang Xiao, Lingyan Liu, Xuewei Li, Mingzhou Li

**Affiliations:** Institute of Animal Genetics and Breeding, College of Animal Science and Technology, Sichuan Agricultural University, Chengdu, 611130 China

**Keywords:** Gene expression, miRNAs, Gene expression, miRNAs

## Abstract

Animals that lived at high altitudes have evolved distinctive physiological traits that allow them to tolerate extreme high-altitude environment, including higher hemoglobin concentration, increased oxygen saturation of blood and a high energy metabolism. Although previous investigations performed plenty of comparison between high- and low-altitude mammals at the level of morphology, physiology and genomics, mechanism underlying high-altitude adaptation remains largely unknown. Few studies provided comparative analyses in high-altitude adaptation, such as parallel analysis in multiple species. In this study, we generated high-quality small RNA sequencing data for six tissues (heart, liver, spleen, lung, kidney and muscle) from low- and high-altitude populations of four typical livestock animals, and identified comparable numbers of miRNAs in each species. This dataset will provide valuable information for understanding the molecular mechanism of high-altitude adaptation in vertebrates.

## Abstract (summary)

**Table:** 

Measurement(s)	miRNA
Technology Type(s)	small RNA sequencing assay
Factor Type(s)	geographic location • altitude • age • weight
Sample Characteristic - Organism	Bos taurus • Bos grunniens • Ovis aries • Sus scrofa • Gallus gallus

Machine-accessible metadata file describing the reported data: 10.6084/m9.figshare.9805424

## Background & Summary

Organisms encounter constant environmental changes, which occurred throughout their lifetime and across generations. As organisms move into a new habitat, natural selection helps facilitating evolution of phenotypes that are better equipped to survive in the new environment. As a classic example of adaptation to a novel environment, high-altitude adaptation involves multifaceted adaptations, including at the level of morphology^1,2^, genetics^3,4^ and physiology^5–7^. Previous studies investigated genetic mechanisms of high elevation adaptation, and identified numerous genes that are under selection. It was reported that persistent changes in gene expression may be induced by long-term environmental changes, and can respond rapidly to sudden external challenges^8^.

microRNAs (miRNAs) are a class of ~22 nucleotide (nt) RNAs that post-transcriptionally regulate gene expression through translational inhibition or mRNA degradation^9^. It was reported that miRNAs play important roles in cold acclimation^10,11^, hypoxia stress^12,13^ and ultraviolet radiation stress^14,15^. For example, miR-32 is induced by cold, and directly targets *transducer of ERBB2, 1* (*Tob1*) to activate p38/MAPK signaling to promote brown adipocyte function and trans-activates white fat browning through *fibroblast growth factor 21* (*FGF21*) in mice^16^. miR-210, a so-called ‘hypoxamir’ regulated by HIF-1α, has been demonstrated to target a variety of genes involved in the cell cycle, mitochondrial metabolism, angiogenesis, DNA damage repair, and cell survival^17^. Thus, it is reasonable to suppose the important roles of microRNAs in high-altitude adaptation.

**Fig. 1:**
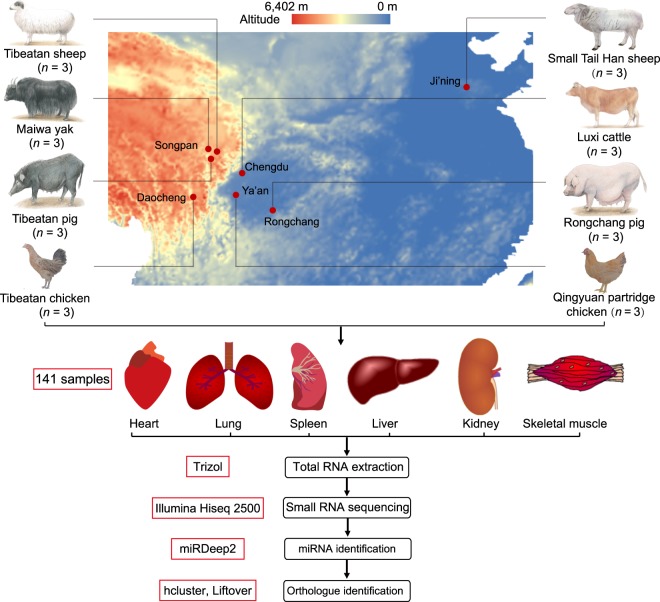
Graphical representation of animal distribution, sample collection and the process of RNA extraction, small RNA sequencing and data analysis.

To reveal the potential roles of miRNAs in high-altitude adaptation, we generated high-quality small RNA sequencing data for six tissues (heart, liver, spleen, lung, kidney, muscle) from high- and low-altitude populations of three species: chicken, sheep and pig. In addition, we also generated small RNA sequencing data from low-altitude cattle and high-altitude yak populations (Fig. 1). Detailed sample information is listed in Table 1. In total, 141 samples were analyzed in this study, including 11 samples that were published^18^. Each population was represented by two or three deep-sequenced biological replicates.

**Table 1:** Basic sample collection information.

Species	Location	Altitude	Age	Weight
Cattle (*Bos taurus*)	Chengdu	500 m	>4 year	~300 kg
Yak (*Bos grunniens*)	Songpan	3,000 m	>4 year	~200 kg
Sheep (*Ovis aries*)	Ji’ning	50 m	>3 year	60–70 kg
Sheep (*Ovis aries*)	Songpan	3,000 m	>3 year	30–50 kg
Pig (*Sus scrofa*)	Rongchang	400 m	>1 year	30–50 kg
Pig (*Sus scrofa*)	Songpan	3,000 m	>1 year	30–50 kg
Chicken (*Gallus gallus*)	Ya’an	500 m	>200 days	2.8–3.2 kg
Chicken (*Gallus gallus*)	Daocheng	3,000 m	>200 days	1.4–1.7 kg

Using a detection threshold of ≥4 reads across >40% samples in each species, we detected 2,036 mature miRNAs. Briefly, we identified comparable amounts of mature miRNA genes and precursor miRNAs for each species (Table 2). The unbiased miRNA annotation for each species prominently improved the uniformity of microRNA count in domesticated animals compared with miRbase21.0, which facilitated comparative analysis of miRNA evolution in domesticated animals.

**Table 2:** The number of miRNAs identified in this study.

Species	Number of miRNAs identified in this study				miRBase 21.0
	Known	Conserved	Novel	Total	
Chicken	267 (169)	37 (32)	17 (15)	321 (216)	740/(994)
Pig	347 (202)	72 (62)	36 (30)	455 (294)	382 (411)
Cattle/Yak	517 (327)	43 (39)	31 (28)	591 (394)	808 (793)
Sheep	147 (91)	225 (160)	50 (38)	422 (289)	106 (153)

Note: The number in front indicates the number of mature miRNA sequences, and the number in parentheses indicates the number of precursor miRNA sequences.

**Fig. 2:**
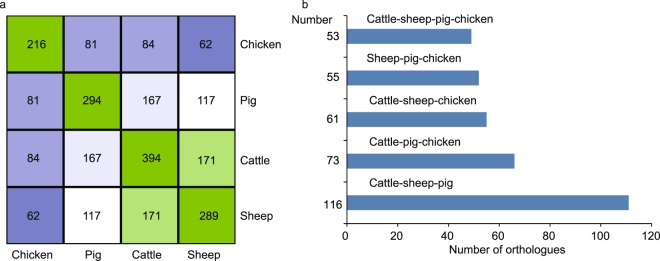
The number of one to one miRNA orthologues identification across four species. (**a**) Number of miRNA orthologues identified in each pair of species. (**b**) Number of miRNA orthologues across three species or four species.

In addition, one-to-one orthologous miRNA genes were also identified based on evolutionary relationships. Overall, we identified 53 orthologues among vertebrates, 116 orthologues in Artiodactyla and 171 orthologues in ruminants (Fig. 2), consistent with a previous study which reported ~100 miRNA orthologues in mammals^9^. The detailed information of one-to-one orthologous miRNA genes for each pair of species is listed in figshare^19^ (The detailed information about one to one miRNA orthologue, figshare 10.6084/m9.figshare.c.4584113).

## Methods

### Sample collection and RNA isolation

Three adult females for each population of the five species from distinct altitudes were used **(**Table 1**)**. These populations have been confirmed to live at corresponding altitudes for their lifetime. Animals were humanely killed to ameliorate suffering. Upon excision from the corpse, a piece of tissue fragments from six organs including heart, liver, spleen, lung, kidney and skeletal muscle(*longissimus* muscle for pig, cattle, yak and sheep, and *pectoral* muscle for chicken) were immediately placed frozen in liquid nitrogen and then stored at −80 °C until use. Total RNA was extracted using the standard Trizol (Takara, Japan) protocol.

### Small RNA library preparation, sequencing, and miRNA annotation

Small RNA libraries were constructed using the Illumina TruSeq Small RNA Sample Prep kit. Libraries were assessed using the Agilent 2200 TapeStation and sequenced on the Illumina HiSeq. 2500 platforms. Initial bioinformatics analysis (base calling) was performed with CASAVA 1.8 (Illumina, USA) to generate raw reads (in FASTQ form). Next, raw reads were subjected to a series of stringent filters (such as removing low-quality reads, repeated sequences, and adaptor sequences) and processed sequences were then mapped to the corresponding reference genome [i.e., chicken (Galgal4), pig (Suscrofa 10.2), cattle (UMD3.1), goat (CHIR 1.0), and sheep (Oar v3.1)] for each species with stringent criteria (<1 mismatch along the whole sequence) using Bowtie^20^. Then, mapped reads were submitted to miRDeep2.0^21^ to detect miRNAs for each species with default parameters. Mature miRNA sequences of chicken and all annotated mammalian species in miRbase21 were selected as reference. To identify miRNAs with ≥4 reads across >40% samples were retained for further analysis. Notably, our previous study identified comparable numbers of miRNA species and similar expressional patterns in bovine genome and yak genomes^18^, therefore, we selected the bovine genome as the reference genome in this study.

### Orthologous miRNA identifications

To identify miRNA orthologues, we first performed an all-against-all BLATST^22^ analysis of miRNA precursors for a specific evolutionary taxonomy. All BLAT matches with >75% of both precursors aligned, an identity of ≥70% and an e-value < 10^−5^ were retained, retained matches were clustered using hcluster (http://treesoft.svn.sourceforge.net/viewrc/treesoft/trunk/hcluster). Clustered families with only one member for each species were first defined as “natural one-to-one” orthologues. For those families with more than one member in any species, we performed liftOver^23^ of each reference genome on human miRNA coordinates to assistant with extraction of one-to-one orthologues.

## Data Records

Small RNA-seq data of heart and lung for yak/cattle which we published before^18^ were available in the NCBI Gene Expression Omnibus (GEO) under accession number GSE87833^24^, and the small RNA-seq data of 126 remaining samples (excluded four samples with mapping rate <65%) were under accession number GSE124418^25^. The detailed information of 126 samples is uploaded to figshare^19^ (Metadata of samples submitted to the NCBI Gene Expression Omnibus, figshare 10.6084/m9.figshare.c.4584113).

## Technical Validation

### Sequencing quality control

**Fig. 3:**
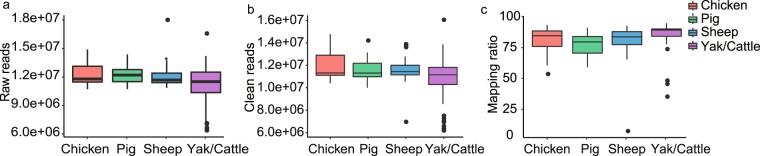
Overview of small RNA-Seq data. (**a**) Distribution of raw reads. (**b**) Distribution of clean reads, and (**c**) mapping rate of each sample for each species.

As shown in Fig. 2, the quality of the small RNA sequencing was checked by analyzing the raw reads, clean reads, and mapping rate for each sample. Briefly, total of 1.66 Gb raw data were obtained. After stringent filtering, 1.60 Gb high-quality reads were remained and an average proportion of 80.89% high-quality reads can be mapped to the respective genomes (Fig. 3 and Sequencing quality control for 141 transcriptomes, figshare 10.6084/m9.figshare.c.4584113). Notably, four samples with mapping rate <65% were excluded to ensure data quality.

### Reproducibility validation

**Fig. 4:**
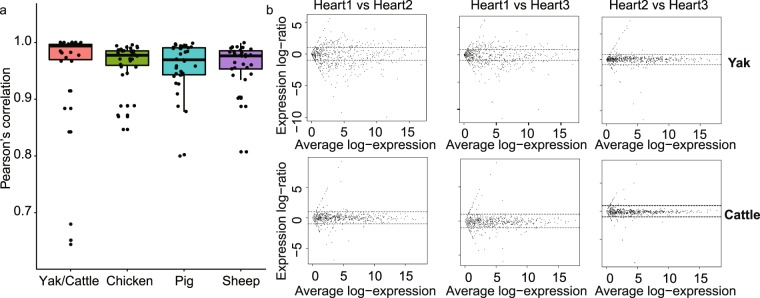
Assessment of reproducibility across biological replicates. (**a**) Pearson’s correlation coefficient of biological replicates for each species. (**b**) MvA plots for examining between-variation of heart in yak/cattle group. The Y-axis represents a log (base 2) fold-change in expression changes and the X-axis indicates the log of the average gene expression level.

To examine the reproducibility of biological replicates, we calculated the Pearson’s correlation for each pair of biological replicates. The majority of biological replicates showed high Pearson’s correlation coefficient (Fig. 4a). Meanwhile, we evaluated between-replicate variation with MvA plots for all pairs. Consistent with Pearson’s correlation analysis, most of biological replicates showed minor variation (Fig. 4b and MvA plots for examining between-replicates variation, figshare 10.6084/m9.figshare.c.4584113). Principal component analysis also showed that the majority biological samples could be grouped together (Principal component analysis of each tissue for each species, figshare 10.6084/m9.figshare.c.4584113). These results highlight the strong experimental confidence of this dataset.

## Usage Notes

Because of its ability to identify novel miRNAs and accurately determine quantitative expression, small RNA sequencing technique has made it possible to obtain large datasets^26^. High-altitude adaptation has recently become a topic of interest, and attracted many specialists in fields such as genetics and molecular biology. We believe that this dataset will provide valuable groundwork for understanding the molecular mechanisms of high-altitude adaptation in vertebrates. These data could also provide an opportunity to comprehensively compare the similarity and difference of the mechanisms underlying high-altitude adaptation across multiple domesticated animals. In addition, our laboratory profiled the mRNA transcriptome for corresponding samples, which facilitates more precise investigation of the interaction between miRNAs and mRNA genes associated with vertebrate high-altitude adaptation^27^ Epigenetic modification vary from one individual to another and could be affected by sex, age, weight, habitat, nutrition and so on, thus it is necessary to be cautious to interpret the mechanism of high altitude adaptation using this dataset.
